# Cortico-cerebral histogenesis in the opossum *Monodelphis domestica*: generation of a hexalaminar neocortex in the absence of a basal proliferative compartment

**DOI:** 10.1186/1749-8104-5-8

**Published:** 2010-03-19

**Authors:** Elisa Puzzolo, Antonello Mallamaci

**Affiliations:** 1 International School for Advanced Studies (SISSA/ISAS), Trieste, Italy

## Abstract

**Background:**

The metatherian *Monodelphis domestica*, commonly known as the South-American short-tailed opossum, is an appealing animal model for developmental studies on cortico-cerebral development. Given its phylogenetic position, it can help in tracing evolutionary origins of key traits peculiar to the eutherian central nervous system. The capability of its pup to regenerate damaged cortico-spinal connections makes it an ideal substrate for regenerative studies. Recent sequencing of its genome and the *ex utero *accessibility of its developing cerebral cortex further enhance its experimental interest. However, at the moment, a comprehensive cellular and molecular characterization of its cortical development is missing.

**Results:**

A systematic analysis of opossum cortico-cerebral development was performed, including: origin of cortical neurons; migration of these neurons from their birthplaces to their final layer destinations; and molecular differentiation of distinct neocortical laminae.

We observed that opossum projection neurons and interneurons are generated by pallial and subpallial precursors, respectively, similar to rodents. A six-layered cortex with a eutherian-like molecular profile is laid down, according to the inside-out rule. However, neocortical projection neurons are generated by apical neural precursors and almost no basal progenitors may be found in the neuronogenic neopallial primordium. In the opossum neocortex, *Tbr2*, the hallmark of eutherian basal progenitors, is transiently expressed by postmitotic progenies of apical precursors prior to the activation of more mature neuronal markers.

**Conclusions:**

The neocortical developmental program predates Eutheria-Methatheria branching. However, in metatherians, unlike eutherians, a basal proliferative compartment is not needed for the formation of a six-layered neuronal blueprint.

## Background

The marsupial South-American short-tailed opossum, *Monodelphis domestica*, is an appealing animal model for developmental studies on cortico-cerebral development for a variety of reasons. First is the phylogenetic position of marsupials, with its implications. Metatherians (or marsupials) are one of the three subclasses of modern mammals, the other two being prototherians (or monotremes) and eutherians (commonly referred to as placentals). Branching between modern sauropsides and mammals took place about 300 million years (My) ago; separations among mammalian subclasses were more recent, 180 My ago for eutherian/metatherian lineages [1], and around 210 My ago for the prototherian lineage [2,3]. Accordingly, marsupials can provide a valuable tool for tracing evolutionary origins of key traits peculiar to the placental central nervous system (CNS). A second reason for our interest is that the opossum pup is able to regenerate connections between neurons of the cerebral cortex and spinal cord damaged by experimental trauma, which makes it an ideal substrate for regenerative studies [4-7]. Third, since the opossum cortex mainly develops after birth, newborns of this species are particularly suitable for early *ex utero *micro-surgical manipulations of such structures [8,9]. Fourth, the complete *M. domestica *genome has recently been sequenced [10], facilitating molecular studies on this model. Important also is that *Monodelphis *is particularly suitable for laboratory studies since it is small, has a short gestational period (2 weeks) and reproduces prolifically throughout the year [8].

Until recently, marsupial cortico-cerebral development has been commonly investigated using classic histology methods [11]. A hexalaminar cortical organitazion, similar to the placental one, has been recognized in *M. domestica *and *Didelphis virginiana *[8,12], and an 'inside-out' gradient of neuronal generation has been assessed in *Trichosurus vulpecula *[13]. Thalamo-cortical connectivity has also been studied by lipophylic dye tracing [14]. However, several features of *Monodelphis *corticogenesis are unknown. The generation times of distinct neocortical laminae have not been determined, knowledge of their molecular diversification is relatively poor, and little is known about where and how distinctive neocortical neuron types are generated. Glutamatergic neurons might largely originate from progenitors within the pallial ventricular zone (VZ), since a basal progenitor compartment, corresponding to the source of the vast majority of neocortical projection neurons in placentals [15-17], appears in the opossum only at late developmental stages [18,19]. Conversely, GABAergic interneurons might arise from the ventral telencephalon, as in other amniota [20-22]. However, both issues have still to be experimentally addressed.

Nowadays, the large body of molecular tools and methodologies used for developmental studies on the cortex of placentals is suitable for addressing cortical histogenesis in marsupials in detail. The availability of *M. domestica *genomic sequence data makes their exploitation even more feasible. Taking advantage of these tools, we tried to fill gaps in our knowledge of opossum corticogenesis, studying the origin of cortical neurons, their laminar differentiation and their migration profiles from periventricular layers to their final layer positions.

## Results

### Molecular differentiation of neurons belonging to distinct neocortical laminae and their radial migration

**Figure 1:**
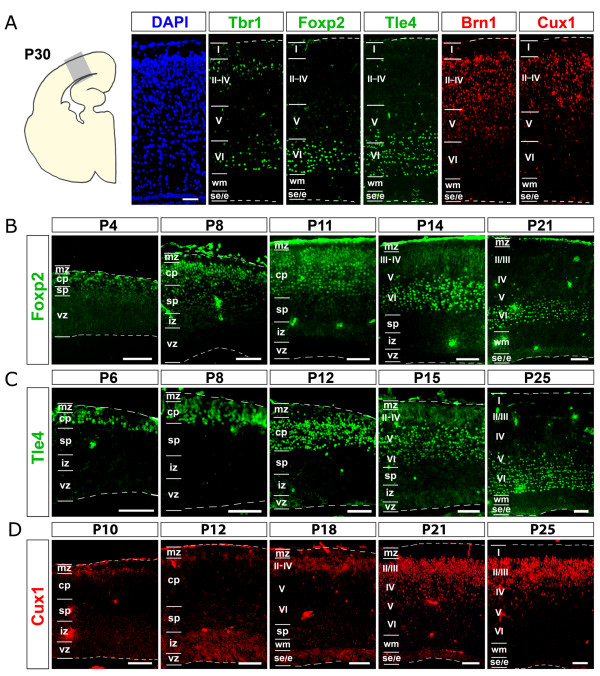
**Expression profiles of layer-specific markers in the opossum neocortex**. **(A) **DAPI staining and Tbr1, Foxp2, Tle4, Brn1 and Cux1 immunofluorescence, on adjacent P30 mid-frontal neocortical sections. **(B-D) **Time course immunoprofiling of Foxp2, Tle4 and Cux1 on mid-frontal neocortical sections, from P4 to P25. Abbreviations: I, II, III, IV, V, VI refer to cortical layers; cp, cortical plate; e, ependyma; iz, intermediate zone; mz, marginal zone; ppl, preplate; se, subependymal zone; sp, subplate; vz, ventricular zone; wm, white matter. Scale bars: 100 μm.

The placental neocortex is formed of six layers, each expressing a well defined set of molecular markers [23]. To assay possible conservation of the neocortical laminar profile between placentals and marsupials, we looked at the distribution of a selection of these markers in the opossum neocortex at postnatal day (P)30, a developmental age at which radial neuronal migration seems to be largely completed (Figure 1A). Tbr1, expressed by mouse subplate, marginal zone and layer VI (as well as, to a lesser extent, layers III and II) [24], was detectable in the opossum in two stripes of cells. The deeper, corresponding to layer VI, included stronger labeled neurons; the more superficial, corresponding to layers II and III, displayed less intense immunoreactivity. Only a few weakly labelled Tbr1^+ ^cells were found in layer I, if any. Foxp2 and Tle4, markers of deep layers in placentals, were both confined to the deep grey matter of the opossum. As expected, Tle4, expressed by mouse layers VI and V, displayed a wider radial domain compared to Foxp2, restricted to murine layer VI only [24]. Conversely, Cux1 and Brn1, markers of upper layers in placentals, were both confined to the superficial grey matter. Again as expected, Brn1, also labeling a subset of layer V neurons in the mouse [25], displayed a wider radial domain compared to Cux1, a marker of layers II to IV only [26].

Next, to reconstruct the temporal order of layer generation, we followed two complementary approaches. First, we assayed the time-course of expression of selected laminar markers, Tle4, Foxp2, and Cux1 for cortical plate (CP) as well as Calretinin and *Reelin *(*Reln*) mRNA for preplate (PPL). Second, we performed systematic bromodeoxyuridine (BrdU) pulse-chase birthdating analysis.

Time-course analysis of laminar markers gave results similar to the mouse. There were, however, some differences. As for Foxp2, no neocortical signal was detectable at P0, when a few strongly labeled cells were conversely present in basal ganglia (data not shown). A Foxp2 signal appeared in lateral neocortex by P4. At P8, this signal spread to the entire cortical plate, becoming progressively confined to the deepest part of it at later developmental ages (Figure 1B). A similar profile was displayed by the deep cortical plate marker Tle4 (data not shown and Figure 1C). As for Cux1 (Figure 1D), two weak and hardly detectable signals were found at P10, in periventricular layers and in a few cells in the upper cortical plate. These signals were stronger at P12, and, by P18, the abventricular expression domain became wider than the periventricular one. By P25, Cux1^+ ^cells were tightly clustered in the most superficial cortical plate and no more Cux1 was detectable near the ventricle.

**Figure 2:**
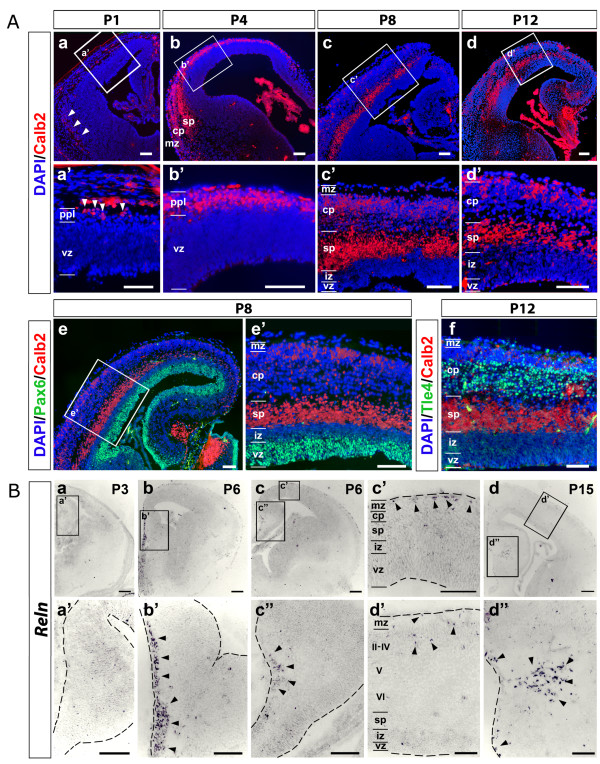
**Dynamics of Calretinin and Reelin expression in the developing opossum telencephalon**. **(Aa-d) **Time course immunoprofiling of Calretinin (Calb2) from P1 to P12. **(Aa'-d') **Magnifications of boxed areas in (Aa-d). **(Ae-f) **Comparisons among distributions of Calretinin, Pax6 (Ae,e') and Tle4 (Af). **(B) ***In situ *hybridization of *Reelin *(*Reln*) mRNA on coronal sections of P3, P6 and P15 opossum telencephalons. (Ba'-d") are magnifications of boxed areas in (Ba-d). Abbreviations: II-IV, V, VI refer to cortical layers; cp, cortical plate; iz, intermediate zone; mz, marginal zone; ppl, preplate; sp, subplate; vz, ventricular zone. Scale bars: 100 μm. Arrowheads in A point to Calb2^+ ^cells within the ppl (a,a'); arrowheads in B point to *Reln*^+ ^neurons at around the septo-pallial border (b,b'), in the neocortical marginal zone (c,c'), the cortical hem (c,c",d,d"), the neocortical outer cortical plate (d,d').

Calretinin (Calb2), expressed by mouse subplate and Cajal-Retzius cells, was detectable in the opossum telencephalon throughout neuronogenesis (Figure 2A). At P1, Calretinin^+ ^cells were mainly localized in the ventral telencephalon and, within the cortex, restricted to the most marginal-lateral part of it (Figure 2Aa,a', arrowheads). At P4, positive cells were throughout the cortical plexiform layer (PPL), including the hippocampus; within the lateral cortex, the Calretinin^+ ^domain was split into two stripes, the more superficial including the marginal zone (MZ), the deeper corresponding to the subplate (SP) (Figure 2Ab,b'). This SP domain, relatively wider compared to the mouse one (Figure 2Ac,c',d,d'), persisted up to P18 (Additional file 1A), disappearing around P20 (Additional file 1B, C). This domain was separated from the ventricular Pax6 domain by the interposed intermediate zone (IZ; Figure 2Ae,e') and abutted the layer VI-V Tle4 domain on its marginal side (Figure 2Af). However, Calretinin was not restricted to PPL and its derivatives. From P4 until P18, weaker labeled Calretinin^+ ^cells were detectable within the outmost cortical plate, where the last generated neurons settle (Figure 2Ab,c',d'; Additional file 1A). Starting from P20 and, better, at P30, a distinct, areally restricted, strong Calretinin expression domain was evident a few cell rows deeper to the MZ (Additional file 1B-F).

As for Reelin, this glycoprotein is a hallmark of Cajal-Retzius cells in the mouse, where additional *Reln*^+ ^cells can also be found in layers IV-V of the late cortical plate [27-29]. We studied *Reln *expression in the opossum by a riboprobe corresponding to exons 1 to 12 and found a spatio-temporal profile similar to the mouse one (Figure 2B). No *Reln *signal was detectable in the pallium at P3, that is, just before the appearance of the CP (Figure 2Ba,a'). Three days later, however, at P6, numerous *Reln*^+ ^cells were found in the neocortical MZ (Figure 2Bc,c'), as well as in the marginal cingulate cortex (Figure 2Bb,b') and in the stratum lacunosum-moleculare of the hippocampus (Figure 2Bc,c"), that is, near two of the main birthplaces of Cajal-Retzius cells described in placentals [29,30]. This expression pattern was retained at least up to P15. At this age *Reln*^+ ^cells within the neocortical MZ were much more sparse and additional *Reln*^+ ^elements were detectable within the developing neocortical CP (Figure 2Bd-d").

**Figure 3:**
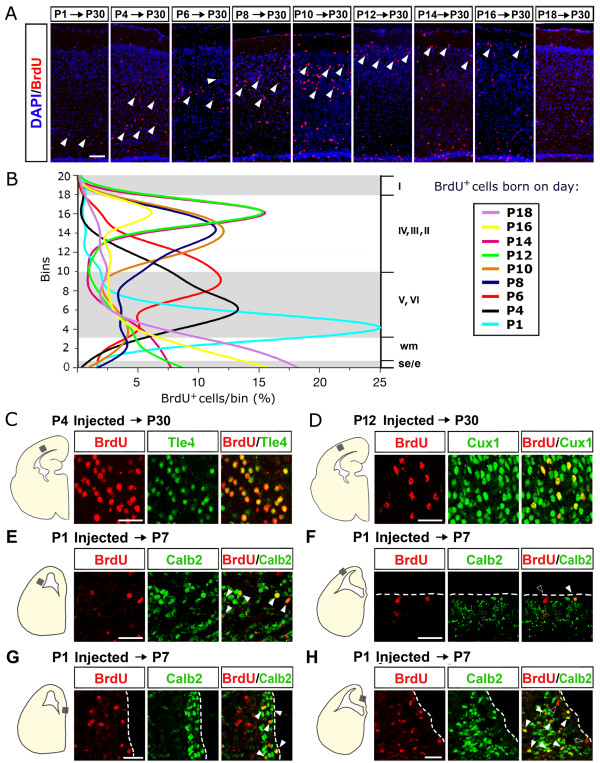
**Bromodeoxyuridine birthdating of opossum neocortical neurons**. **(A) **BrdU immunoprofiling of mid-frontal neocortical sections from opossums injected with a single pulse of BrdU at the ages of P1, P4, P6, P8, P10, P12, P14, P16 and P18, and fixed at P30. Arrowheads point to grey matter BrdU^+ ^cells. **(B) **Diagramatic representation of BrdU^+ ^cells sampled in (A): the cortical wall is divided into 20 equally spaced bins, numbered from ventricular to marginal; radial extension of cortical laminae is indicated by white/grey shading; plots representing percentages of BrdU^+ ^cells falling into each bin, for each injection time. **(C-H) **Colocalization of layer-specific markers, Tle4 (C), Cux1 (D), Calb2 (E-H), with BrdU injected at P4 (C), P12 (D) and P7 (E-H), respectively. Solid arrowheads in (E-H) point to Calb2^+^/BrdU^+ ^cells; empty arrowheads in (F, H) point to Calb2^-^/BrdU^+ ^cells. Abbreviations: I, II, III, IV, V, VI refer to cortical layers; e, ependyma; se, subependymal zone; wm, white matter. Scale bars: 100 μm in (A); 40 μm in (C-H).

To reconstruct the temporal order of layer generation, we also performed systematic BrdU pulse-chase birthdating analysis. By this approach, cells which were in S-phase at the time of BrdU injection and exited the cell cycle immediately afterwards, remained heavily labeled and, as such, easily traceable upon completion of their radial migration. We injected opossum pups at different developmental ages (P1, P4, P6, P8, P10, P12, P14, P16, and P18) with a single pulse of saturating BrdU and left them to develop until the age of P30, when all neurons have reached their final laminar position (as shown in Figures 1 and 2). We recovered their brains and analyzed the cortices by BrdU immunofluorescence (Figure 3A). To compare radial distribution and laminar identities of BrdU^+ ^cells in distinct brains, we divided the cortical wall into 20 equally spaced bins, numbered from ventricular to marginal, and on this framework reported the approximative radial extension of distinct cortical laminae: layer I (evaluated by loose DAPI staining), layers II-IV (by Cux1 immunofluorescence), and layers V-VI (by Tle4 immunofluorescence). Then, for each injection time, we counted BrdU^+ ^cells, calculated the percentage of them falling into each bin and plotted the data. Finally, we superimposed the resulting curves, obtaining a synopsis of the whole radial migration process (Figure 3B).

We found that neocortical neurons were generated in a wide temporal window, mainly from P1 to P14. Cells born at P16 reached superficial layers only to a limited extent, suggesting that at that age neurogenesis was over, and P18 cells prevalently remained beneath the CP. Deep cortical plate neurons were prevalently born between P1 and P6, and upper cortical plate neurons between P8 and P14. Colocalization of Tle4 and Cux1 with BrdU in P30 animals injected at P4 and P12, respectively, confirmed this conclusion (Figure 3C, D). As the SP is not anymore distinguishable at P30, we assayed the date of birth of its neurons in distinct, dedicated experiments. By administering P1 pups with BrdU and recovering their brains at P7 and P12, BrdU^+^/Calretinin^+ ^cells were detectable beneath the CP, especially in lateral cortex (Figure 3E and data not shown), suggesting that the SP is mainly generated around birth. Finally, consistent with *Reln *data, P1 BrdU-pulsed/Calretinin^+ ^cells, corresponding to presumptive Cajal-Retzius cells [31], were also detectable at P7 in the neocortical MZ (Figure 3F), the marginal cingulate cortex (Figure 3G), and the hippocampal stratum lacunosum-moleculare (Figure 3H).

In conclusion: in the opossum, neocortical neuronogenesis begins at the time of birth and ends 2 weeks later, at P14 to P16, and radial migration is completed by P25; the molecular laminar profile is very similar in marsupials and placentals; and after PPL splitting, cortical plate neurons are laid down in both mammalian subclasses according to the same 'inside-out' rule.

### Does a basal progenitor compartment exist in the opossum?

In placentals, neocortical projection neurons are prevalently generated by basal progenitors or intermediate progenitor cells, which lie around the pallial subventricular zone-VZ border and divide far from the ventricular surface [16,17]. In the developing opossum cortex, a subventricular zone is not morphologically distinguishable [8]. A basal proliferative compartment has been reported recently [19], although only after neuronogenesis completion (Figure 3B). We systematically readdressed this issue by assaying mitosis distribution and immunoprofiling distinct neuronogenic progenitors throughout the neuronogenetic window and beyond.

**Figure 4:**
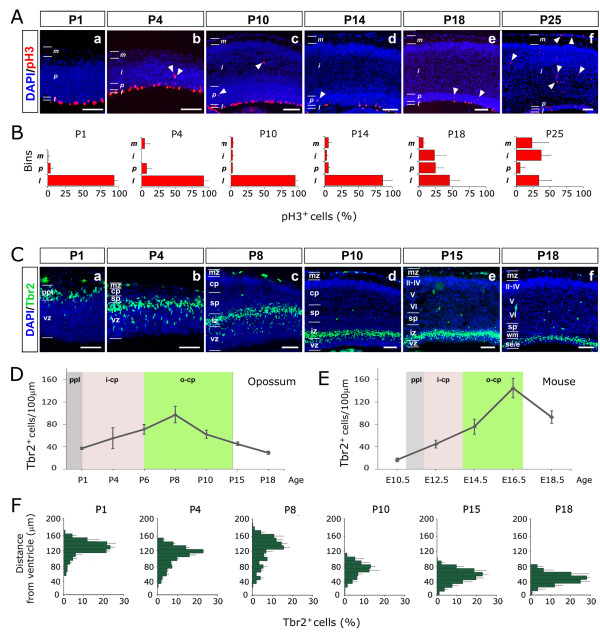
**Dynamics of phospho-histone3 and Tbr2 expression in the developing opossum cortex**. **(A) **Phospho-histone3 (pH3) immunoprofiling of mid-frontal neocortical sections of P1 to P25 opossums. Immunopositive cells are prevalently aligned near the ventricle; solid arrowheads point to rare abventricular pH3^+ ^mitotic cells. **(B) **Diagrammatic representation of pH3^+ ^cells sampled in (A). **(C) **Tbr2 immunoprofiling of mid-frontal neocortical sections of P1 to P18 opossums. **(D-F)**. Linear densities (D) and radial distributions (F) of Tbr2^+ ^cells sampled in (C). (E) Linear densities of Tbr2^+ ^cells in the mouse. Grey, pink and green shading in (D, E) demarcate peak neuronogenesis windows for primordial plexiform layer (ppl), inner cortical plate (i-cp) and outer cortical plate (o-cp), respectively. Abbreviations: II-IV, V, VI refer to cortical layers; cp, cortical plate; e, ependyma; iz, intermediate zone; mz, marginal zone; ppl, preplate; se, subependymal zone; sp, subplate; vz, ventricular zone; wm, white matter. Scale bars: 100 μm in (A, C).

First, we studied the distribution of cortical progenitors undergoing mitosis at distinct radial positions, starting from P1 up to P25, by scoring the mitotic marker phospo-histone 3 (pH3) (Figure 4A). For each developmental age, we divided the cortical wall into four unequally spaced bins, *l *(luminal, including the two ventricular most cell rows), *p *(periventricular, corresponding to the densely packed zone over the ventricle minus the *l *belt), *i *(intermediate, corresponding to the region between *p *and MZ), and *m *(marginal, corresponding to the MZ), and plotted the percentages of pH3^+ ^cells falling in each of them (Figure 4B). We observed that the vast majority of pH3^+ ^cells were aligned along the ventricular surface, as proper apical progenitors, whereas only a few of them were scattered elsewhere. Up to P14, a few mitoses could be found in bin *p*, corresponding to the main zone where basal progenitors divide in placentals (Figure 4Ab, B). These mitoses were prevalently localized near the cortico-striatal notch, in the presumptive paleocortical sector (Additional file 2). The frequency of *p *mitoses rose considerably after P18. Starting from this age, numerous abventricular mitoses could also be found in bins *i *and *m *(Figure 4Af, B), as described for placental MZ glial progenitors [32].

As a complementary approach, we looked for cortical expression of the T-box transcription factor Tbr2, a hallmark of basal progenitors in placentals [33]. We found numerous Tbr2^+ ^cells at all stages under examination, from P1 to P18 and later (Figure 4C and data not shown). On 10-μm-thick sections, their linear frequency gradually rose from 37 ± 2 cells/100 μm at P1 to 97 ± 14 cells/100 μm at P8 (P1 to P8 is the time window when PPL, deep CP and part of superficial CP are generated), subsequently declining to 45 ± 3 cells/100 μm at P15 (that is, just after the end of neuronogenesis) and even less at P18 (Figure 4D). Remarkably, this time course mirrored that of murine Tbr2^+ ^cells (Figure 4E), which, however, were more numerous and reached their peak linear density slightly later, close to the end of neuronogenesis. Finally, as for radial distribution, modal distance between Tbr2^+ ^cells and ventricular surface varied in the opossum from about 120 to 140 μm at P1 to P8, to 60 μm at P15. In synthesis, Tbr2^+ ^cells were detectable throughout the neuronogenetic window, prevalently clustered around the border between the VZ and IZ, but also scattered within the VZ, exactly like placental basal progenitors [33] (Figure 4F).

So, based on pH3 data, a basal proliferative compartment does not seem to exist within the neuronogenic neocortical field (the frequency of abventricular mitoses starts to increase just around the end of neuronogenesis). Based on Tbr2 expression, a compartment molecularly similar to the basal one of placentals can be distinguished, interposed between ventricular precursors and abventricular neurons. How to solve this conundrum? Rather than being due to the absence of proliferative activity, the paucity of abventricular pH3^+ ^cells might reflect a very slow cell cycle progression in front of a short duration of M phase [34,35] (our unpublished observations), possibly leading to an underestimation of proliferative population. To rule out this possibility, we looked for presumptive basal cells in S phase, which would reasonably be several times more frequent than the corresponding M phase ones [34,35]. So, we pulsed P4 to P10 opossum pups with BrdU, fixed their brains and looked for cortical Tbr2^+ ^cells also immunoreactive for BrdU. Such cells were extremely rare, (N_(Tbr2+BrdU+)_/N_(Tbr2+) _= 0 at P4, 0 at P6, 3.3 ± 1.6 × 10^-3 ^at P10), and the vast majority of Tbr2^+ ^cells lay above the BrdU^+ ^ones (Additional file 3A). Moreover, as expected, Tbr2^+ ^cells were even more rarely positive for the mitotic pH3 marker (N_(Tbr2+pH3+)_/N_(Tbr2+) _= 0 at P4, 0 at P6, 4.4 ± 2.9 × 10^-4 ^at P10, 5.4 ± 2.7 × 10^-4 ^at P14; Additional file 3B). These data strongly suggest that, in marsupials, Tbr2^+ ^cells are not proper basal progenitors, but mainly represent a subventricular postmitotic compartment, interposed between intermitotic apical progenitors and postmitotic abventricular neurons.

**Figure 5:**
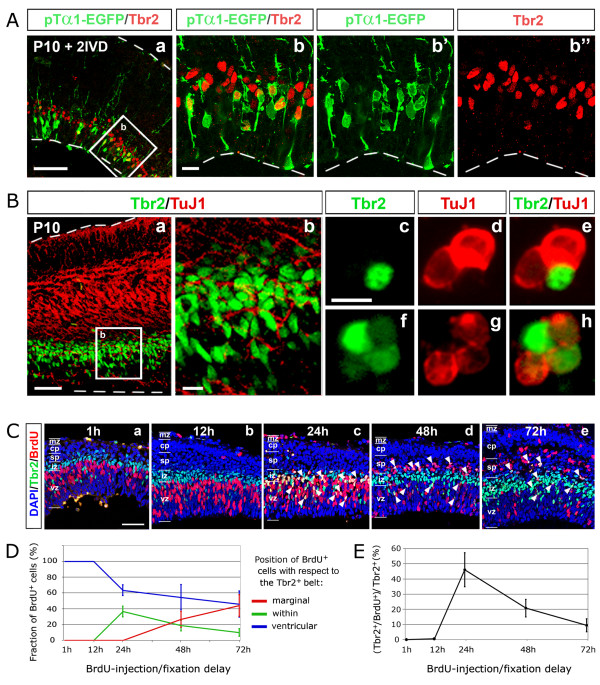
**Origin and fate of Tbr2^+ ^cells**. **(A, B) **Confocal Tbr2/enhanced green fluorescent protein (EGFP) (Aa-b") and Tbr2/β-tubulin (Ba, b) immunoprofiling of opossum cerebral cortex, dissected out at P10, acutely electroporated with a pTα1-EGFP plasmid and kept in *in vitro *culture for 48 h. Magnifications of boxed areas in (Aa) and (Ba) are shown in (Ab-b") and (Bb), respectively. (Bc-h) Colocalization of Tbr2 and β-tubulin on acutely dissociated cells from P10 opossum cortex. **(C) **Confocal Tbr2/BrdU immunoprofiling of neocortical coronal sections from opossum pups, pulsed with BrdU at P6 and sacrificed after different times: 1 h, 12 h, 24 h, 48 h and 72 h. Arrowheads in (Cc-e) point to cells immunoreactive for both BrdU and Tbr2. **(D) **Relative radial distribution of BrdU^+ ^cells sampled in (C) compared to the Tbr2^+ ^belt. **(E) **Time course of percentages of Tbr2^+^cells sampled in (C) also immunoreactive for BrdU. Abbreviations: cp, cortical plate; iz, intermediate zone; mz, marginal zone; sp, subplate; vz, ventricular zone. Scale bars: 100 μm in (A-C); 20 μm in (Ab, Bb, c).

To corroborate this interpretation, we further investigated the origin and fate of Tbr2^+ ^cells. In rodents, Tbr2^+ ^cells are generated by a subset of neuronally committed apical progenitors, called 'pin-like' cells or short neural precursors, which, facing the ventricular cavity and not contacting the pial surface, can be distinguished from radial glial cells for specific firing of the α1-tubulin promoter (pTα1^+ ^cells) [36,37]. We electroporated a pTα1-enhanced green fluorescent protein (EGFP) construct into the P10 opossum cortex and fixed electroporated brains 2 days later, after a final pulse (60 minutes) of BrdU. After verifying their vitality and cytoarchitectonic integrity (by scoring radial distribution of BrdU^+ ^and Tbr2^+ ^cells; Additional file 4), we finally looked for GFP^+^/Tbr2^+ ^cells (Figure 5A). We found many of them (Figure 5Ab-b"), confirming that opossum Tbr2^+ ^cells derive from apical progenitors, as in rodents. We then compared the distribution of Tbr2 and the neuronal marker β-tubulin on coronal slices of P10 opossum brains (Figure 5B). Tbr2^+ ^cells lay below the main β-tubulin domain, but a substantial overlap between the two antigens was detectable as well (Figure 5Ba,b): in particular, around 30% of acutely dissociated neocortical cells expressing Tbr2 were also positive for β-tubulin (Figure 5Bc-h), consistent with the hypothesis that Tbr2 is transiently expressed before the activation of neuron-specific markers.

Finally, to get a better temporal resolution of the developmental process under examination, we pulsed P6 pups with BrdU, fixed their brains after different times (from 6 h up to 72 h), and immunoprofiled BrdU^+ ^cells, performing time-course analysis of their radial distribution (Figure 5C, D). Comparing profiles obtained at different times, BrdU^+ ^cells seemed to move along a wave, from the ventricular side towards the marginal aspect of the cortical wall. In particular, up to 12 h, all BrdU^+ ^cells lay deep to the Tbr2^+ ^belt (Figure 5Cb); starting from 24 h, some of them were detectable within this belt (Figure 5Cc); at 48 h, about one-third of them were above it (Figure 5Cd); finally, at 72 h, most had overcome the Tbr2^+ ^belt, so that only a few remained near the ventricle (Figure 5Ce). Remarkably, the percentage of Tbr2 cells also immunoreactive for BrdU displayed a biphasic trend, arising from about 0% at 12 h up 46% at 24 h and subsequently declining to less than 10% at 3 days (Figure 5E). In synthesis, neural progenitors exiting the cell cycle leave the VZ and activate Tbr2 18 ± 6 h after the last DNA synthesis; 1 day later, the same cells massively move to more marginal positions, where they downregulate Tbr2, while activating neuron-specific markers (Figure 5Bc-h).

### The apical proliferative compartment and its dynamics

**Figure 6:**
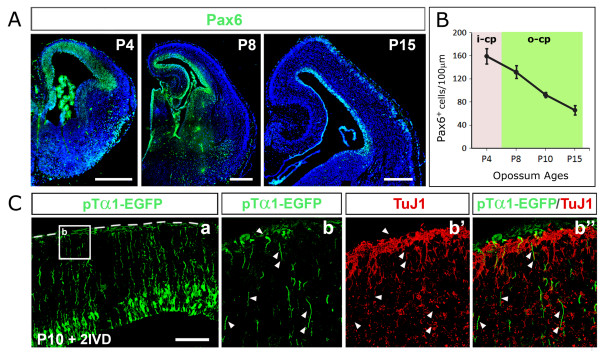
**Analysis of the apical progenitor compartment**. **(A) **Pax6 immunoprofiling of a selection of coronal sections from P4 to P15 opossum telencephalons. **(B) **Linear densities of Pax6^+ ^cells sampled in (A). **(C) **EGFP/β-tubulin immunoprofiling of opossum cerebral cortex, dissected out at P10, acutely electroporated with a pTα1-EGFP plasmid and kept in *in vitro *culture for 48 h. Magnifications of the boxed area in (Ca) are shown in (Cb-b"). Arrowheads in (Cb-b") point to pial processes of electroporated cells, immunoreactive for EGFP, but not for β-tubulin. Abbreviations: i-cp, inner cortical plate; o-cp, outer cortical plate. Scale bars: 400 μm in (A); 100 μm in (C).

Previous results indicate that the apical progenitor compartment is the place where pallial projection neurons are generated. To reconstruct the dynamics of this compartment, we performed time-course analysis of its hallmark, Pax6 [33]. As in placentals, this homeoprotein was specifically expressed in the pallial VZ throughout neuronogenesis (Figure 6A). On 10-μm-thick sections, linear frequencies of Pax6^+ ^cells were 160 ± 16 cells/100 μm at P4, 132 ± 4 cells/100 μm at P8, 92 ± 8 cells/100 μm at P10, and 66 ± 11 cells/100 μm at P15 (Figure 6B). These values were slightly lower than the corresponding mouse ones (185 ± 16 cells/100 μm at embryonic day (E)12.5, 156 ± 13 cells/100 μm at E14.5, and 96 ± 13 cells/100 μm at E16.5; data not shown), although the two temporal progressions were basically very similar. These results suggests that decreased neuronal density and reduced thickness of the opossum cortex [38], rather than reflecting a change of the apical proliferative compartment size, mainly originates from the absence of a basal, transient amplifying population.

Moreover, in placental neocortex, the Pax6^+ ^compartment is not homogeneous. It includes neural stem cells with the morphology of radial glial cells and pTα1^+ ^neuronally committed progenitors, having lost their contact with the pial surface. It is noteworthy that, upon electroporation of the pTα1-EGFP transgene into the opossum cortex, fluorescent cells displayed morphology of proper radial glial cells, extending from the ventricular edge to the pial surface (Figure 6Ca). Moreover, despite the time elapsed after electroporation, pial processes and end-feet of many of these cells were still not immunoreactive for neuron-specific β-tubulin, ruling out that they were neurons (Figure 6Cb-b"). This means that, in marsupials, either pTα1 fires in radial glial cells, or retraction of the pial process by neuronally committed progenitors is delayed compared to rodents.

### Distribution and generation of cortical GABAergic cells

**Figure 7:**
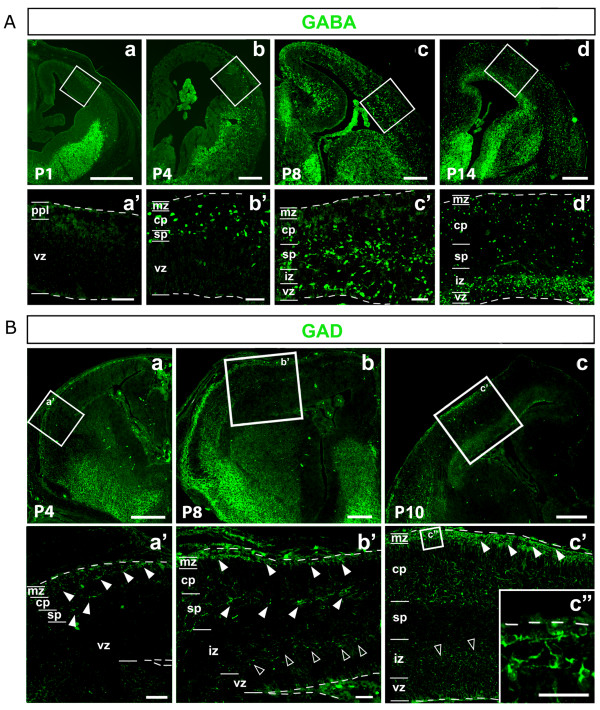
**Distribution of GABA and glutamate-decarboxylase immunoreactivity in developing opossum cortices**. **(Aa-d) **Distribution of GABA^+ ^cells on frontal sections of P1 to P14 cortices. **(Aa'-d') **Magnifications of boxed regions in (Aa-d). **(Ba-c) **Distribution of glutamate-decarboxylase (GAD)^+ ^cells on frontal sections of P4, P8 and P10 cortices. **(Ba'-c') **Magnifications of boxed regions in (Ba-c). GAD^+ ^cells are evident in the marginal zone and the subplate since P4 (Ba',b', solid arrowheads). At P8, an additional reactivity is detectable in proliferative layers (Bb', empty arrowheads). Finally, at P10, both marginal zone and periventricular GAD signals are strengthened (Bc',c") and additional GAD^+ ^cells are present throughout the cortical plate (Bc'). Abbreviations: cp, cortical plate; iz, intermediate zone; mz, marginal zone; sp, subplate; vz, ventricular zone. Scale bars: 400 μm in (Aa-d,Ba-c); 50 μm in (Aa'-d', Ba'-c").

In the mouse, GABAergic neurons are generated within ventral forebrain and reach the cortex by tangential migration, according to a well characterized spatio-temporal pattern [39,40]. First, interneurons enter the cortex at around E12.5, and the migratory wave reaches the mid-neocortex at E13.5 and then the hippocampus by E15.5. Early migration is mainly superficial; by E13.5 a robust periventricular migratory route appears, becoming predominant at later stages. In order to establish developmental correspondences between mouse and opossum, we systematically scored the distribution of GABA immunoreactive cells in the developing post-natal telencephalon of the marsupial. From P1 up to P14, a huge number of GABA^+ ^cells were detected within the ventral telencephalon. Here, they were early confined to abventricular layers, and later also detectable near the ventricle (Figure 7Aa-d). Cortical distribution was more complex. At P1 only a diffuse and light immunoreactivity was detectable throughout the marginal pallium (Figure 7Aa'), and rare GABA^+ ^somata were localized in presumptive paleocortex (Figure 7Aa and data not shown). At P4 a substantial number of GABA^+ ^cells were present in the abventricular half of both paleo- and neocortex, but not in the archicortex (Figure 7Ab,b'). At P8, abventricular GABA^+ ^cells were present throughout the cortex, including the marginal hippocampus; ventricular GABA^+ ^cells were conversely limited to paleo- and neocortex (Figure 7Ac,c'). Finally, at P14, GABA^+ ^cells were detectable throughout the cortex, clustered in a narrow periventricular belt and more loosely distributed elsewhere (Figure 7Ad,d'). Remarkably, the spatio-temporal distribution of glutamate-decarboxylases 65 and 67 (GAD65 and GAD67) was consistent with the GABA pattern (Figure 7B).

The apparently higher frequency of GABA^+ ^cells within the opossum cortical VZ, compared to the mouse, suggested that, in marsupials, at least some of these cells might be locally generated. To test this hypothesis, we assayed the capability of early cortical tissue, not containing any GABA^+ ^cells and isolated from ventral forebrain, to generate such cells *in vitro*. We dissected out cerebral cortices from P1 opossum brains, cultured them *in vitro *for 8 days in the presence of saturating iododeoxyuridine (IdU), exposed then to a final pulse of BrdU before fixation (90 minutes), and finally immunoprofiled them for GABA, IdU and BrdU (Additional file 5Aa). We found cells expressing the three antigens in different combinations. Numerous BrdU^+ ^cells were detectable, suggesting that, before fixation, cultures were still healthy (Additional file 5Ab). Among GABA^+ ^cells, some were IdU^- ^(Additional file 5Ac-c", empty arrowheads). These cells were reasonably generated before the dissection and acquired their mature GABA^+ ^phenotype in culture. However, a large fraction of GABA^+ ^cells was also IdU^+ ^(Additional file 5Ac-c", solid arrowheads), suggesting that they could have been locally generated by cortical progenitors that underwent their final DNA synthesis during *in vitro *culturing.

To confirm such a conclusion, we re-addressed this issue *in vivo*. We pulsed P5 pups with BrdU, fixed their brains 24 h later and finally looked for neocortical BrdU^+^/GAD^+ ^or BrdU^+^/GABA^+ ^cells as an index of local interneuron generation. We reasoned that 24 h should be a sufficiently long time delay to allow newborn neurons of the GABAergic lineage to switch robust GAD and GABA immunoreactivities on and sufficiently short to avoid ventrally generated interneurons entering the cortex. As expected, we found many BrdU^+^/GAD^+ ^or BrdU^+^/GABA^+ ^cells in basal ganglia. However, only very few of them were detectable in neocortex (Additional file 5B). All this suggests that, in marsupials, as in rodents, most interneurons are generated outside the cortex, in the basal telencephalon. The relatively high frequency of IdU^+^/GABA^+ ^cells we found in cortical explants may have two explanations. The few interneurons generated in cultured cortical explants accumulated over as many as 8 days and, due to experimental detachment of cortex from basal ganglia, were poorly diluted by those coming from the subpallium. Moreover, in a large fraction of cases, IdU was probably not uptaken by GABAergic-lineage pallial precursors during pre-neuronogenic DNA synthesis, but by progenies of subpallial precursors (already present in the cortical explant at the time of its removal), during post-mitotic DNA repair. These two mechanisms are not mutually exclusive and may largely account for apparent inconsistencies between the results of the *in vitro *and *in vivo *approaches.

### Post-neuronal histogenesis: generation of astrocytes and oligodendrocytes

In rodents, gliogenesis follows neuronogenesis and that happens in the developing opossum cortex as well. To assess astrocytogenesis progression, we looked at the distribution of the Glial fibrillary acid protein antigen (Gfap) from P8 (that is, the middle of the neuronogenic window) onward. A periventricular signal detected from the beginning was restricted to hippocampus at P8 (Additional file 6Aa), and spread throughout the cortical field at P12 (Additional file 6Ab). This signal presumptively corresponds to the soma of radial glial cells, which, in primates, share this marker with true astrocytes [41,42]. An abventricular Gfap signal associated with cells with the morphology of astrocytes could be found only later, starting from P18 to P20 (data not shown; Additional file 6Ac-f). These cells were detectable in the MZ, the grey matter and white matter. A subset of them coexpressed the astrocyte marker S100β (Additional file 6Ag-i").

The differentiation of oligodendrocytes was studied by monitoring immunoreactivity for the marker O4 [43]. A strong signal was found at both analyzed ages, P40 and P60, restricted to white matter, internal capsule and hippocampal commissure (Additional file 6Ba,b; *M. domestica *has no corpus callosum [44]).

## Discussion

In the present study we found that the cortico-cerebral neuronal complement is generated in the opossum pup between P1 and P16, molecular diversification of neurons belonging to distinct laminae largely resembles that of placentals, and migration of cortical plate neurons follows the 'inside-out' rule. We demonstrated that opossum neocortical projection neurons are almost entirely generated by apical progenitors, and that Tbr2, the hallmark of placental basal progenitors, is only transiently expressed by opossum post-mitotic elements, prior to the activation of neuron-specific genes. Moreover, we showed that such absence of a basal transient amplifying population is the main reason for the reduced thickness and decreased neuronal density, distinguishing the marsupial from the placental cortex [38,45] despite conserved intracolumnar upper/lower layer neuron ratios [19]. As for GABAergic neurons, we found that whereas many are born in the subpallium, as in rodents [39,40], and invade the cortex by P4, a small fraction of them is also generated within cortical periventricular layers. Finally, as in placentals, cortical histogenesis continues with astrocytogenesis (from P18 onward) and ends up with oligodentrocytogenesis (around P40 and later).

We found that molecular diversification of the cortical plate is highly conserved between marsupials and placentals, with Foxp2 and Tle4 restricted to deeper layers and Brn1 and Cux1 mainly confined to upper layers. This suggests that the neocortical hexalaminar profile arose before the branching between these two subclasses, about 180 My ago [46]. Minor differences were observed in derivatives of PPL, namely the phylogenetically most ancient component of our cortex [47]. Based on Calretinin immunostaining, the opossum SP appeared quite prominent, as previously assessed by simple histological inspection [8], far thicker than in rodents [48]. Cells expressing *Reln*, a hallmark of Cajal-Retzius neurons, were detectable beneath the pia mater, in both neo- and archicortex, like in placentals. However, their appearance did not predate the splitting of the preplate, as happens in rodents [48]. We reconstructed rules governing marsupial neocortical lamination by following two complementary experimental approaches. First, we compared the mature distribution of neocortical laminar markers with the radial settling profile of neurons generated at different developmental times, as assessed by BrdU pulse-chase analysis. Second, we performed systematic time-course expression analysis of a selection of these markers, from their appearance to the end of the lamination process. Both approaches indicated that, in *M. domestica*, radial neuronal migration takes place in a way similar to placentals. Like in rodents [27-29], such a process is reasonably promoted by the glycoprotein Reelin, released by Cajal-Retzius cells and, later, by some CP neurons. This protein seems, however, to be dispensable for PPL splitting, which apparently occurs in the absence of detectable expression of its mRNA.

Beyond the study of laminar differentiation and radial migration, we paid special attention to the origin of cortical neurons, both glutamatergic and GABAergic. In placentals, glutamatergic neurons are generated within the dorsal telencephalon by two periventricular proliferative compartments, the apical and the basal, the former confined to the VZ, the latter mainly lying in the subventricular zone [17,49]. The existence of a basal compartment in the opossum was controversial [18]. A recent study reports that such a compartment is actually present in developing marsupial brains, suggesting that it emerged in the eutherian-metatherian lineage [19]. In the opossum, however, basal proliferative activity arises only around the end of cortical neuronogenesis (Figure 3B) and thus contributes little to the generation of the neocortical neuronal complement. We reanalyzed this topic by an integrated approach, including scoring of pH3^+ ^mitoses and Tbr2^+ ^cells, immunoprofiling of acutely administrated BrdU and pTα1-driven EGFP, as well as BrdU pulse-chase analysis. We found that, in the opossum, cells expressing Tbr2 do not form a transient amplifying population, but represent a postmitotic transitional compartment, passed through by neuroblasts in the process of switching Pax6 off and activating neuron-specific β-tubulin. Interestingly, among sporadic abventricular pH3^+ ^precursors detectable in the cortex at P14, more than four-fifths do not express Tbr2 (Additional file 3B), suggesting that such precursors may belong not to the neuronogenic but to the gliogenic lineage. This scenario is not surprising. Tbr2 has been detected in the developing pallium of other vertebrates missing a basal proliferative compartment, such as Anamnia and birds [50-52]. Moreover, besides corticogenesis, Tbr2 is also expressed in the gastrulating embryo, where it promotes epithelium-to-mesenchyme transition [53]. So this transcription factor, rather than specifying basal progenitor identity in the developing cortex, might play a more general morphogenetic role, inducing apical precursors to leave embryonic epithelia they belong to [16].

Anyway, it has to be recalled that some abventricular pH3^+ ^mitoses can be found in the lateral-most opossum pallium. It is tempting to speculate that such basal proliferative activity might increase the final neuronal output of the small ventricular sector in between neopallial and striatal fields, in charge of generating paleocortex and other latero-ventral derivatives of the amniote telencephalon [54].

Time-course GABA/GAD immunoprofiling of the developing cortex, immuno-characterization of early cortical explants as well as *in vivo *short-term BrdU pulse-chase of GABAergic neurons showed that generation of cortical interneurons in the opossum is mainly confined to ventral telencephalon, like in other Amniota [20-22]. Actually, there is some local cortical interneurono-genesis; however, the amplitude of this phenomenon is modest as in rodents [55], far smaller than in primates [56-58].

## Conclusions

This study provides three main results on the developing opossum cortex. First, projection neurons and interneurons are generetad by pallial and subpallial precursors, respectively, as in eutherians. Second, a neocortex with a eutherian-like molecular profile is laid down, following the inside-out rule, suggesting that the hexalaminar blueprint emerged prior to eutherian-metatherian branching. Third, differently from eutherians, neocortical projection neurons are almost entirely generated by apical neural precursors and basal progenitors hardly contribute to their genesis; Tbr2, the hallmark of eutherian basal progenitors, is transiently expressed by postmitotic progenies of apical precursors prior to the activation of more mature neuronal markers.

**Figure 8:**
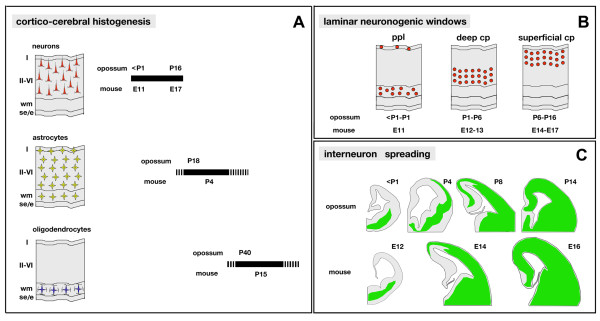
**Opossum and mouse cortico-cerebral histogenesis: a comparison**. **(A) **Radial distribution and approximate temporal generation windows of neurons, astrocytes and oligodendrocytes in the opossum and mouse cerebral cortex. **(B) **Peak generation times for primordial plexiform layer, deep cortical plate and superficial cortical plate in opossum and mouse. **(C) **Temporal profile of interneurons spreading in the developing cerebral cortex of opossum and mouse. Abbreviations: I, II-VI refer to cortical layers; cp, cortical plate; e, ependyma; ppl, primordial plexiform layer; se, subependymal zone; wm, white matter.

Based on temporal progression of the morphogenetic subroutines that were the subject of analysis, a comparative opossum/mouse cortical developmental timetable is sketched in Figure 8.

## Materials and methods

### Animals

Opossums (*M. domestica*) at different postnatal ages were obtained from the colony maintained at the animal house facility of the University of Trieste, Italy. Mice (*Mus musculus*, strain CD1) were purchased from Harlan (San Pietro al Natisone, UD, Italy). Opossums were staged by systematic daily inspection of the colony for newborn litters, P0 corresponding to the day of birth. Mice were staged by timed breeding and vaginal plug inspection. Animal handling and subsequent procedures were in accordance with European laws (European Communities Council Directive of November 24, 1986 (86/609/EEC)) and with National Institutes of Health guidelines. In particular, to harvest opossum brains (from P1 to P40), young animals were killed by decapitation, after hypothermia. When the entire CNS had to be recovered, the animals were alternatively killed by aorta resection, again after hypothermia. In the case of P60 animals, they were terminally anesthetized by urethane and transcardially perfused with 4% paformaldehyde. Mouse embryos (E10.5 to E18.5) were harvested from pregnant dames killed by cervical dislocation.

### Bromodeoxyuridine administration

BrdU was administered to P1 to P18 opossum pups at a dose of 200 μg/g body weight in 0.9% NaCl by subcutaneous injections. During administration, pups were left attached to the mother, previously anesthetized with isofluorane. All injected animals were sacrificed at the age of P30 and their brains used for kinetic studies.

### Organotypic cultures

Two kinds of organotypic brain cultures were used for this study, adherent and floating. Adherent cultures were set up according to the classical Stoppini method [59], with minor modifications. Briefly, P1 opossum neocortices and basal ganglia were dissected, placed on distinct falcon cyclopore PET membranes and kept at the atmosphere-medium interface as follows. After priming (1 h at 32°C in DMEM-F12-Glutamax, 10% FBS/N2, 0.6% glucose, under 95% air/5% CO_2 _atmosphere), membranes were transferred into 12-multiwell plates, each well containing 1.2 ml of Neurobasal-B57-Glutamax, 0.6% glucose (supplemented with Fungizone/Pen/Strept mix), and kept for 8 days at 32°C at the interface between medium and a 95% air/5% CO_2 _atmosphere. For floating cultures, P10 opossum CNSs were dissected and repeatedly washed in DMEM-F12-Glutamax/0.6% glucose/Fungizone/Pen/Strept, paying particular attention to avoid any damage. After further manipulations (DNA injection and electroporation), CNSs were transferred to 50 ml Falcon tubes (one per tube), each containing 15 ml of DMEM-F12-Glutamax/0.6% glucose/N2, 1%FBS/Fungizone/Pen/Strept. Cultures were maintained at 32°C for two days, in the presence of bubbling 95%O_2_/5%CO_2_. In cases of both adherent and floating cultures, to verify brain vitality, a final pulse of BrdU (5 μg/ml of medium) was given 90 minutes before explant fixation.

### Brain electroporation

Electroporation was performed on acutely dissected P10 opossum CNSs, put in a petri dish filled with 0.6% glucose, 1× PBS. An aqueous 1 μg/μl solution of plasmid DNA (1 μl; containing 0.01% fast green dye) was injected into a lateral ventricle using glass capillaries 1B100-3 (World Precision Instruments, Sarasota, FL, USA) prepared using the micropipette puller P-97 (Sutter Instrument Company, Novato, CA, USA). Two parallel, 5-cm spaced, rectangular electrode plates (4 cm × 6 cm) were placed on both sides of the telencephalon and three 100 V pulses (each 100 ms long; interval between consecutive pulses 450 ms) were delivered using an electro-square-porator (BTX 830). Plasmids pTα1-EGFP (kindly provided by E Ruthazer) and pDsRed2-N1 (Clontech, Mountain View, CA, USA) were used for electroporation.

### Histology

CNS specimens (both mouse and opossum, whole brains and explants) were fixed in 4% paraformaldehyde-PBS overnight at 4°C, cryoprotected in 30% sucrose/PBS and cut coronally at 10 μm. Cryosections were mounted on Fischer SuperFrost Plus slides, and subsequently processed for *in situ *hybridization or immunohistochemistry.

To accurately determine the fraction of Tbr2^+ ^cells also expressing β-tubulin, six freshly dissected P10 opossum cortices were pooled and dissociated to single cells by gentle trituration. Cells were resuspended in DMEM-F12, 1% serum, plated onto slides previously covered with 20 μg/ml poly-D-lysine, and left to attach for 1 h at room temperature. Slides were processed for immunofluorescence, as described elsewhere.

### Immunofluorescence

Immunofluorescence was performed as previously described [60], with minor modifications. Generally, prior to use, sections were boiled for 5 minutes in 10 mM pH6 citrate buffer. This step was omitted in the case of β-tubulin, β-tubulin/GFP, GAD and GABA stainings. In the case of BrdU detection, sections were also treated for DNA depurination (2 M HCl, for 15 minutes at room temperature) and then neutralized (in 0.1 M borate buffer, pH 8.5, for 15 minutes at room temperature). For other combined immunofluorescences (BrdU/Tle4, BrdU/Cux1, BrdU/Tbr2, IdU/GABA, BrdU/GAD and BrdU/GABA), the HCl concentration was reduced to 0.2 M. Sections were incubated for 1 h at room temperature under blocking mix (1× PBS, 10% FBS, 1 mg/ml bovine serum albumin, 0.1% Triton X100) and then incubated at 4°C overnight with primary antibody in blocking mix.

Primary antibodies used were as follows: mouse monoclonal anti-BrdU (clone B44, Becton Dickinson, Franklin Lakes, New Jersey, USA), 1:50; rat anti-BrdU (clone ICR1, Abcam, Cambridge, UK), 1:500; goat anti-Brn1 (Santa Cruz Biotechnology, Inc., Santa Cruz, CA, USA), 1:30; mouse monoclonal anti-Calretinin (clone M7245, Dako, Glostrup, Denmark), 1:50; rabbit anti-Cux1 (Santa Cruz), 1:30; rabbit anti-Foxp2 (Abcam), 1:1,500; rabbit anti-Gaba (Sigma-Aldrich Corp., St. Louis, MO, USA), 1:8,000; rabbit anti-GAD (Sigma), 1:1,000; rabbit anti-Gfap (Dako), 1:500; chicken anti-GFP (Abcam), 1:800; mouse monoclonal anti-O4 (clone O4, R&D Systems Inc., Minneapolis, MN, USA), 1:600; rabbit anti-Pax6 (Abcam); 1:300; rabbit anti-pH3 (Upstate Biotechnology Inc, Lake Placid, NY, USA), 1:600; mouse anti-pH3 (Cell Signaling Technologies Inc, Danvers, MA, USA), 1:100 (limited to pH3/Tbr2 colocalization assays); rabbit anti-S100β (Dako), 1:200; rabbit anti-Tbr1 (kindly provided by Robert Hevner), rabbit anti-Tbr2 (Abcam), 1:500; rabbit anti-Tle4 (Santa Cruz), 1:30; mouse anti-neuron-specific class III β-tubulin (clone Tuj1, Covance, Emeryville, CA, USA), 1:500.

Finally, immunoreactivity was revealed after 2-h incubation with secondary Alexa antibodies, 488 and 594 (Molecular Probes, Emeryville, CA, USA), 1:400.

### *In situ *hybridization

Non-radioactive *in situ *hybridization was performed as previously described [61], with minor modifications. The probe used corresponded to exons 1 to 12 of the *M. domestica Reelin *(*Reln*) coding region (Ensembl mdo-chr.8: nt 155064879-154744337).

### Imaging and confocal microscopy

Fluorescent labeled sections were imaged and analyzed using a fluorescent Nikon (Tokyo, Japan) Eclipse 80i microscope and a DS-2MBWC digital microscope camera. Confocal photos were taken by a TCS SP2 Leica confocal microscope; they were generally collected as 1.0-μm-thick Z-stacks, and as 3.0-μm-thick Z-stacks in the case of pTα1-EGFP electroporated cells. All images were processed using Adobe Photoshop CS3 software.

### Sample sizing

Unless otherwise stated, each experiment was performed at least in triplicate. In cases of laminar birthdating (Figure 3), per each BrdU pulsing time, three mid-frontal 800-μm-wide neocortical sectors, from four cortices were profiled. pH3^+ ^cell counting was performed in similarly, but throughout the neocortical field (Figure 4A, B). The same applies to Tbr2^+ ^(Figure 4C-F), Tbr2^+^/BrdU^+ ^(Figure 5C) and Pax6^+ ^(Figure 6A) cell counting, but restricted to 200-μm-wide parietal sectors. Error bars in Figures 4, 5 and 6 represent standard deviations. For Tbr2^+^/BrdU(1 h)^+ ^and Tbr2^+^/pH3^+ ^counting (Additional file 3), at least three animals per age were analyzed (only two for Tbr2^+^/pH3^+ ^counting at P6) and not less than 600 (at P4), 1,600 (at P6), 3,000 (at P10) and 3,000 (at P14) Tbr2^+ ^cells were scored for each brain; Tbr2^+^/BrdU(1 h)^+ ^and Tbr2^+^/pH3^+ ^data are reported in the main text as average ± standard error of the mean. Finally, the prevalence of Tbr2^+ ^elements among abventricular pH3^+ ^cells at P14 was calculated by collecting and scoring about 15 abventricular pH3^+ ^cells per brain, from three distinct brains.

## Abbreviations

BrdU: bromodeoxyuridine; CNS: central nervous system; CP: cortical plate; DMEM: Dulbecco's modified Eagle's medium; FBS: fetal bovine serum; GABA: gamma-aminobutyric acid; GAD: glutamate-decarboxylase; Gfap: Glial fibrillary acid protein antigen; E: embryonic day; EGFP: enhanced green fluorescent protein; IdU: iododeoxyuridine; IZ: intermediate zone; MZ: marginal zone; My: million years; P: postnatal day; PBS: phosphate-buffered saline; pH3: phospo-histone 3; PPL: preplate; SP: subplate; VZ: ventricular zone.

## Competing interests

The authors declare that they have no competing interests.

## Authors' contributions

EP performed the experiments. EP and AM designed the experiments, analyzed data and wrote the manuscript.

## Supplementary Material

Additional file 1 **Late expression of Calretinin in the opossum cortex**. **(A-F) **Calretinin (Calb2) immunoprofiling of coronal sections from P18 to P30 opossum cortexes. Solid arrowheads point to immunopositive cells in the outer cortical plate at P18 (A), and double arrowheads demarcate the deeper, areally restricted expression domain visible starting from P20 onward (C-F). Empty arrowheads highlight the absence of Calb2^+ ^cells in more superficial rows of the CP at P20 and later (B, C, F). Abbreviations: e, ependyma; hi, hippocampus; mz, marginal zone; se, subependymal zone; wm, white matter; II, III, IV, V, VI are cortical layers. Scale bar: 100 μm. Click here for file

Additional file 2 **Abventricular mitotic pH3^+ ^cells in the lateral opossum pallium**. **(A-D) **Opossum telencephalons from P4 to P30. **(A'-D') **Magnifications of boxed areas in (A-D). Solid arrowheads point to abventricular mitotic pH3^+ ^cells within the lateral-most cortex. Scale bar: 300 μm (A-D); 100 μm (A'-D'). Click here for file

Additional file 3 **Mutual periventricular distribution of Tbr2 and proliferation markers in the developing opossum cortex**. **(A) **Comparative Tbr2/BrdU immunoprofiling of P4, P6 and P10 opossum neocortices, pulsed by BrdU 1 h before fixation. **(B) **Comparative Tbr2/pH3 immunoprofiling of selected P4, P6, P10 and P14 opossum neocortical sectors, harboring rare abventricular pH3^+ ^precursors. Empty arrows in (B) point to places occupied by Tbr2^-^pH3^+ ^elements. Scale bar: 50 μm. Click here for file

Additional file 4 **Cytoarchitectonic integrity of opossum cortical explants**. **(A) **Distribution of BrdU and Tbr2 immunoreactivity in the cortex of an opossum brain explanted at P10, electroporated by pTα1-EGFP, kept in floating culture for 48 h and terminally administered with a 60-minute pulse of BrdU. Scale bar: 100 μm. Click here for file

Additional file 5 **Generation of opossum cortical GABA^+ ^and GAD^+ ^cells**. **(Aa) **Strategy for assessing birthplaces of cortical GABA^+ ^cells *in vitro*, on cortical explants. **(Ab-c") **BrdU/IdU/GABA immunoprofiling of radial sections of neo-/archi-cortical explants, dissected out at P1, kept in culture for 8 days in the presence of IdU and terminally administered with BrdU. Empty arrowheads in (Ac-c") point to GABA^+^/IdU^- ^cells, presumptively born before tissue dissection; solid arrowheads point to GABA^+^/IdU^+ ^cells, presumptively generated by cortical progenitors that underwent their final DNA synthesis *in vitro*. **(Ba, Bd) **GAD/BrdU and GABA/BrdU immunoprofiling of P6 telencephalons, from opossum brains pulsed by BrdU 24 h before fixation. **(Bb-b", Bc-c") **Magnifications of boxed regions in (Ba); **(Be-e", Bf-f") **magnifications of boxed regions in (Bd). Arrowheads in (Bb", Bc") point to cortical and basal GAD^+^/BrdU^+ ^cells, respectively. **(Bb"', Bc"') **Magnifications of boxed regions in (Bb", Bc"), where large immunoreactivity spots are detectable around the nucleus of GAD^+ ^cells, from both cortex and ganglia. Arrowheads in (Be", Bf") point to double positive GABA^+^/BrdU^+ ^cells. **(Be"', Bf"') **Magnifications of boxed regions in (Be", Bf"). Abbreviations: BG, basal ganglia; CX, cortex. Scale bars: 200 μm in (Ba, Bd); 50 μm in (Ab-c", Bb-b", Bc-c", Be-e", Bf-f"). Click here for file

Additional file 6 **Gliogenesis in the opossum cortex**. **(Aa-f) **Gfap immunoprofiling of coronal sections from P8 to P60 cortices. Arrowheads in (Aa) and in (Ab) point to Gfap^+ ^presumptive radial glial cells, within P8 hippocampus and P12 cortical periventricular layers, respectively. Arrows in (Ac-f) indicate Gfap^+ ^cells with astrocyte morphology. **(Ag-i") **Combined Gfap/S100b immunoprofiling of P20 to P30 cortices, showing specific colocalization of these two antigens within more mature astrocytes, in cortical plate (CP) and marginal zone (MZ). **(Ba, b) **Immunoprofiling of P40 to P60 mid-frontal cortical sections for the oligodendrocyte-specific marker O4: an intense staining may be found in white matter (WM), internal capsule (ic) and hippocampal commissure (hc). Scale bars: 100 μm in (Aa-f); 50 μm in (Ag-i); 200 μm in (B). Click here for file
