# Supplementary figures and images for: Cortico-cerebral histogenesis in the opossum Monodelphis domestica: generation of a hexalaminar neocortex in the absence of a basal proliferative compartment
Source: Neural Dev. 2010 Mar 19;5:8. doi: 10.1186/1749-8104-5-8 (PMC2859365; doi:10.1186/1749-8104-5-8)

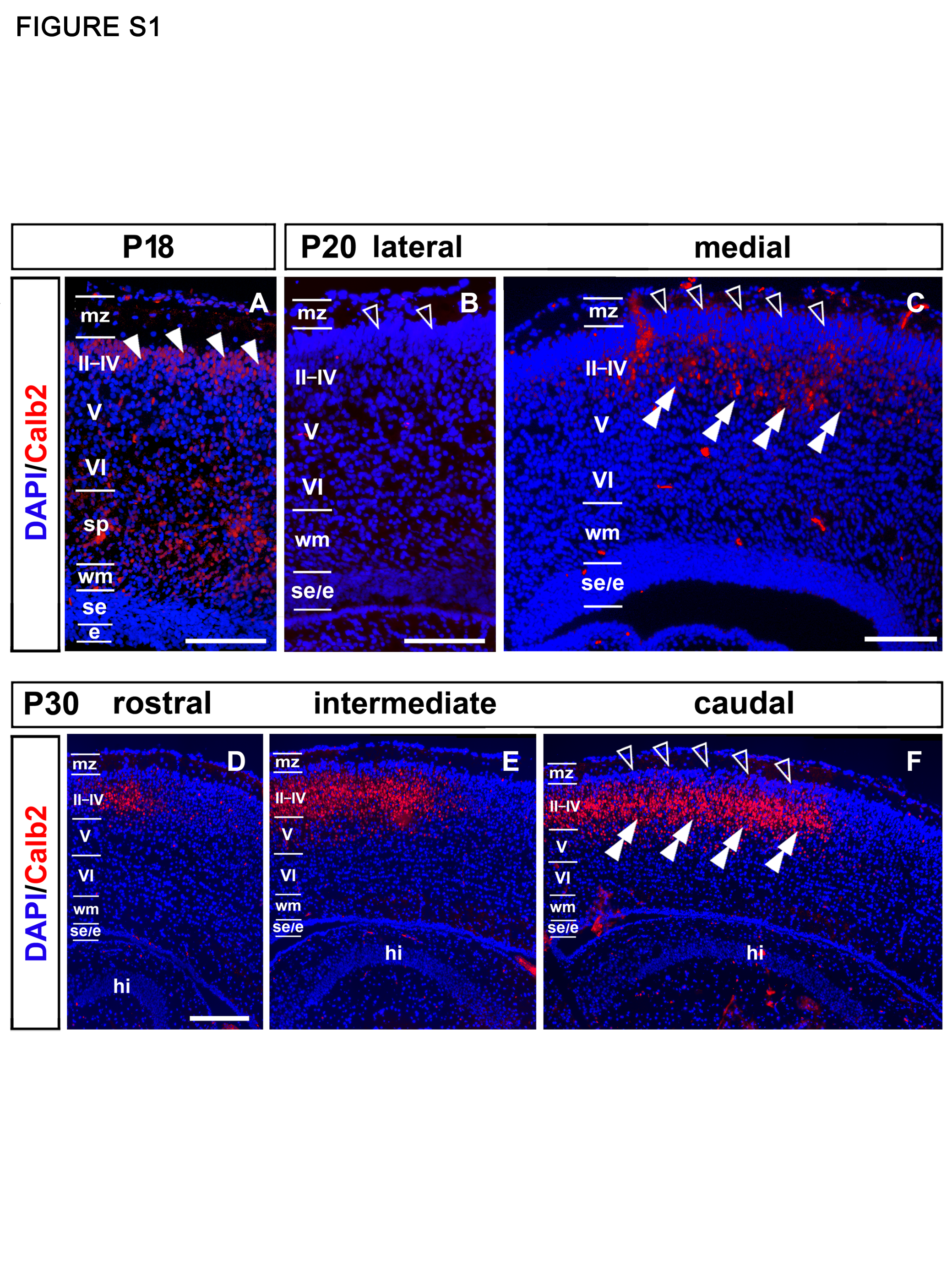

Supplement: Additional file 1 — Late expression of Calretinin in the opossum cortex. (A-F) Calretinin (Calb2) immunoprofiling of coronal sections from P18 to P30 opossum cortexes. Solid arrowheads point to immunopositive cells in the outer cortical plate at P18 (A), and double arrowheads demarcate the deeper, areally restricted expression domain visible starting from P20 onward (C-F). Empty arrowheads highlight the absence of Calb2+ cells in more superficial rows of the CP at P20 and later (B, C, F). Abbreviations: e, ependyma; hi, hippocampus; mz, marginal zone; se, subependymal zone; wm, white matter; II, III, IV, V, VI are cortical layers. Scale bar: 100 μm. [file 1749-8104-5-8-S1.TIFF]

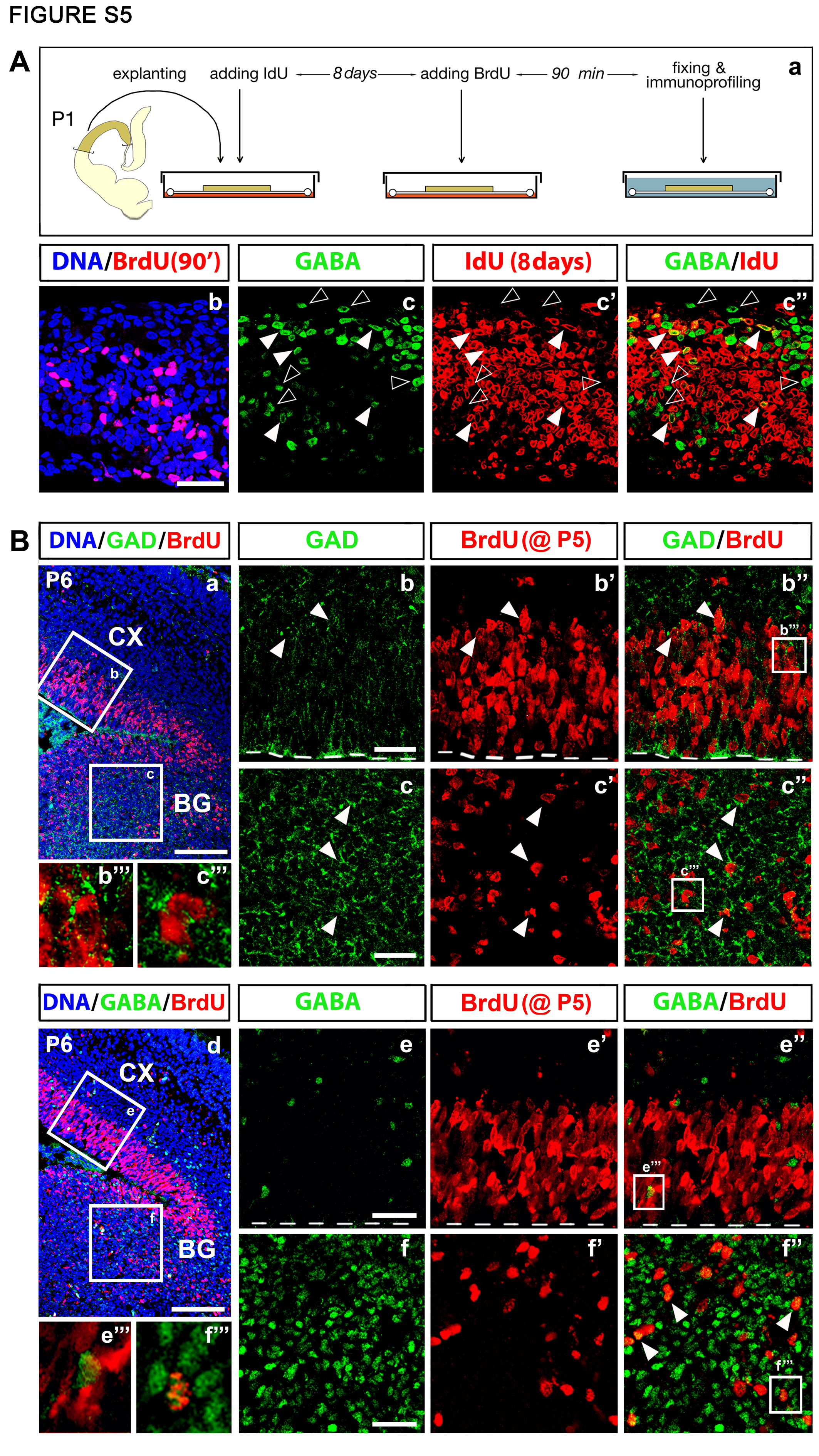

Supplement: Additional file 5 — Generation of opossum cortical GABA+ and GAD+ cells. (Aa) Strategy for assessing birthplaces of cortical GABA+ cells in vitro, on cortical explants. (Ab-c") BrdU/IdU/GABA immunoprofiling of radial sections of neo-/archi-cortical explants, dissected out at P1, kept in culture for 8 days in the presence of IdU and terminally administered with BrdU. Empty arrowheads in (Ac-c") point to GABA+/IdU- cells, presumptively born before tissue dissection; solid arrowheads point to GABA+/IdU+ cells, presumptively generated by cortical progenitors that underwent their final DNA synthesis in vitro. (Ba, Bd) GAD/BrdU and GABA/BrdU immunoprofiling of P6 telencephalons, from opossum brains pulsed by BrdU 24 h before fixation. (Bb-b", Bc-c") Magnifications of boxed regions in (Ba); (Be-e", Bf-f") magnifications of boxed regions in (Bd). Arrowheads in (Bb", Bc") point to cortical and basal GAD+/BrdU+ cells, respectively. (Bb"', Bc"') Magnifications of boxed regions in (Bb", Bc"), where large immunoreactivity spots are detectable around the nucleus of GAD+ cells, from both cortex and ganglia. Arrowheads in (Be", Bf") point to double positive GABA+/BrdU+ cells. (Be"', Bf"') Magnifications of boxed regions in (Be", Bf"). Abbreviations: BG, basal ganglia; CX, cortex. Scale bars: 200 μm in (Ba, Bd); 50 μm in (Ab-c", Bb-b", Bc-c", Be-e", Bf-f"). [file 1749-8104-5-8-S5.TIFF]

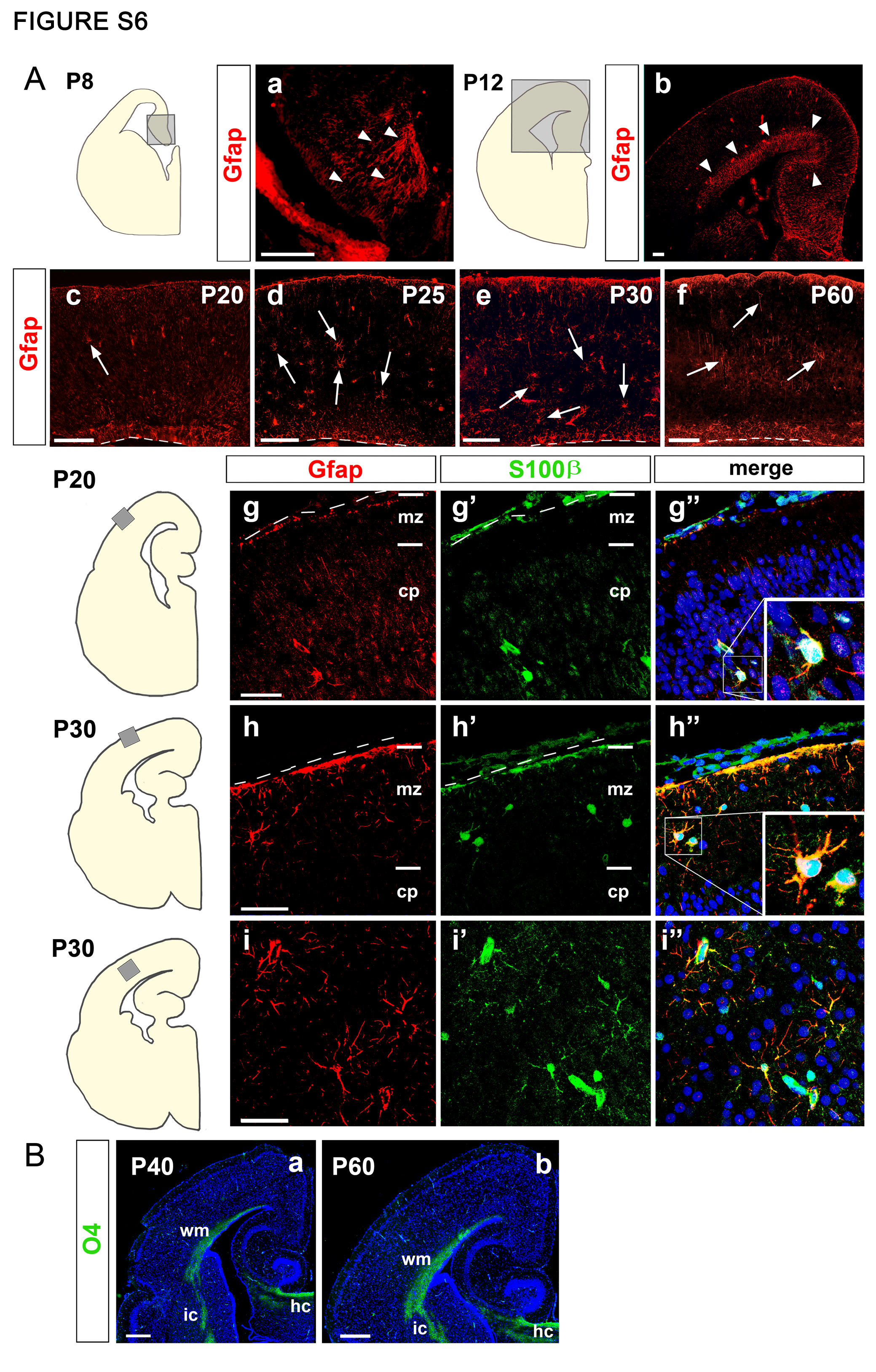

Supplement: Additional file 6 — Gliogenesis in the opossum cortex. (Aa-f) Gfap immunoprofiling of coronal sections from P8 to P60 cortices. Arrowheads in (Aa) and in (Ab) point to Gfap+ presumptive radial glial cells, within P8 hippocampus and P12 cortical periventricular layers, respectively. Arrows in (Ac-f) indicate Gfap+ cells with astrocyte morphology. (Ag-i") Combined Gfap/S100b immunoprofiling of P20 to P30 cortices, showing specific colocalization of these two antigens within more mature astrocytes, in cortical plate (CP) and marginal zone (MZ). (Ba, b) Immunoprofiling of P40 to P60 mid-frontal cortical sections for the oligodendrocyte-specific marker O4: an intense staining may be found in white matter (WM), internal capsule (ic) and hippocampal commissure (hc). Scale bars: 100 μm in (Aa-f); 50 μm in (Ag-i); 200 μm in (B). [file 1749-8104-5-8-S6.TIFF]
